# Reliable and accurate diagnostics from highly multiplexed sequencing assays

**DOI:** 10.1038/s41598-020-78942-7

**Published:** 2020-12-10

**Authors:** A. Sina Booeshaghi, Nathan B. Lubock, Aaron R. Cooper, Scott W. Simpkins, Joshua S. Bloom, Jase Gehring, Laura Luebbert, Sri Kosuri, Lior Pachter

**Affiliations:** 1 Department of Mechanical Engineering, California Institute of Technology, Pasadena, CA USA; 2 Octant Inc., Emeryville, CA USA; 3 Department of Human Genetics, University of California, Los Angeles, Los Angeles, USA; 4 Department of Genome Sciences, University of Washington, Seattle, WA USA; 5 Division of Biology and Biological Engineering, California Institute of Technology, Pasadena, CA USA; 6 Department of Computing and Mathematical Sciences, California Institute of Technology, Pasadena, CA USA

**Keywords:** Infectious diseases, Computational biology and bioinformatics

## Abstract

Scalable, inexpensive, and secure testing for SARS-CoV-2 infection is crucial for control of the novel coronavirus pandemic. Recently developed highly multiplexed sequencing assays (HMSAs) that rely on high-throughput sequencing can, in principle, meet these demands, and present promising alternatives to currently used RT-qPCR-based tests. However, reliable analysis, interpretation, and clinical use of HMSAs requires overcoming several computational, statistical and engineering challenges. Using recently acquired experimental data, we present and validate a computational workflow based on kallisto and bustools, that utilizes robust statistical methods and fast, memory efficient algorithms, to quickly, accurately and reliably process high-throughput sequencing data. We show that our workflow is effective at processing data from all recently proposed SARS-CoV-2 sequencing based diagnostic tests, and is generally applicable to any diagnostic HMSA.

## Introduction

Reliable, scalable, low-cost testing for SARS-CoV-2 is paramount for reducing infection rates and controlling the current pandemic^1^. Currently, SARS-CoV-2 tests are primarily based on RT-qPCR, however, several groups have recently proposed massively parallelized diagnostic assays based on high-throughput sequencing that hold the promise of greatly increased throughput, reduced cost, and improved sensitivity^2−4^. While the proposed diagnostics differ in implementation details, they share several key features:

- (Synthetic) sequence barcodes known as samples indices are associated with samples and are recovered by sequencing.
- (Biological) sequences associated with genes, including viral genes, control genes, or spike-ins, are recovered by sequencing.
- Sequenced sample indices and biological sequences are associated with each other.

These assays bear resemblance to multiplexed barcoding technologies used for single-cell RNA-seq^5−7^, and as a result, the bioinformatics challenges that must be overcome in analyzing the data are similar.

**Figure 1:**
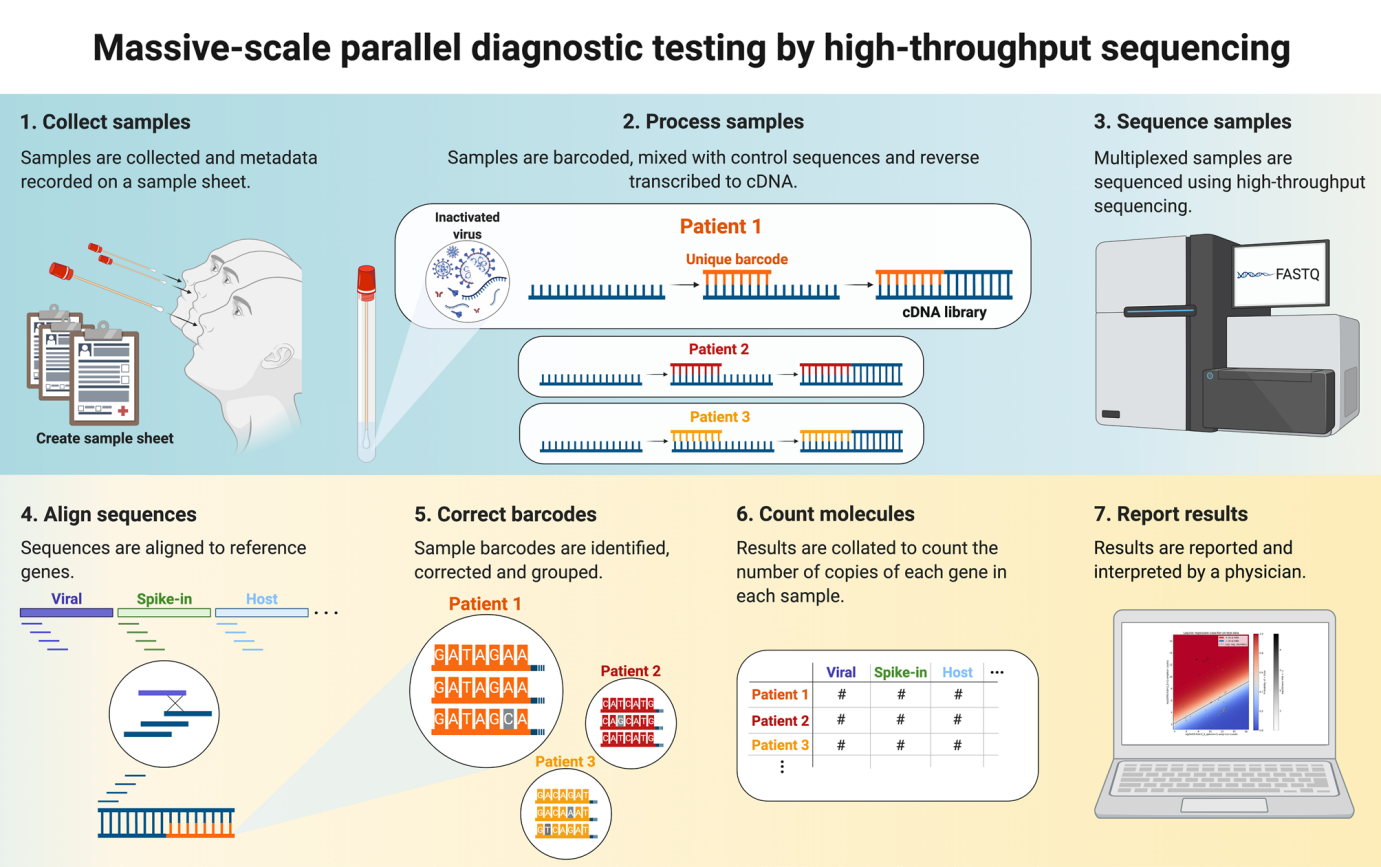
Massively parallel diagnostic testing by high-throughput sequencing. Workflow of a high-throughput sequencing based diagnostic test. (1) Samples are collected and prepared. (2) Samples are barcoded and amplified. (3) Multiplexed samples are pooled and sequenced using a high-throughput sequencer. 4) Sequencing data is aligned to a set of genes, (5) sample indices are error corrected, (6) counts are computed, and (7) diagnostic results are obtained.

Processing of the data requires association of the biological sequences with their genes of origin, error correction of the sample indices and collation of sequences associated with a single sample to count the number of molecules from each gene that have been observed (Fig. 1). Finally, the infection status for each sample must be determined from the gene abundance estimates per sample.

It is crucial that the software used to report infection status for each sample is reliable, well-documented, portable, and open source. These features of diagnostic software instill confidence in healthcare providers and patients, and ensure transparency and reproducibility in a setting where software errors can be deadly. Additionally they make it easier to adapt, improve, and expand on the capabilities of the software to handle novel use cases. Current software programs used for sequencing-based diagnostics, such as bcl2fastq, do not satisfy these crucial requirements, despite being used in FDA approved diagnostics^8,9^.

To address these shortcomings, and to overcome the challenges required for SARS-CoV-2 sequencing-based diagnostics, we adapted the RNA-seq and single-cell RNA-seq tools kallisto^10^ and bustools^11,12^ to HMSA analysis, and coupled them in a workflow we designate “kallisto|bustools” (Supplementary Note). These tools are portable, well-documented, open source, and have a low computational footprint making them usable on a wide variety of architectures. In addition, we developed a testing framework to report infection status, and we validate our results with complementary methods. Our software is freely available under the permissible BSD-2 open source license, and we show that it can be used for SwabSeq^2^, a technology based on Octant’s RNA amplicon sequencing platform; LAMP-seq^3^, which relies on LAMP^13^; covE-seq^14^, which targets the SARS-CoV-2 E gene; or TRB-seq^4^, which is a targeted BRB-seq^15^ variant. The short running time and low memory footprint of the software allow low-cost logistical solutions to data analysis in the clinical setting.

### Results

To validate our workflow, we analyzed 307,494,992 SwabSeq reads (see Methods). This dataset consisted of two 384-well plates each with a titration series of viral RNA from two companies, Twist and ATCC, for a total of 768 uniquely barcoded samples. HEK293 lysate, nasopharyngeal (NP) lysate, and controls were included in all of the wells of each plate. The first plate was used to test primers bound to the SARS-CoV-2 N gene and the second plate was used to test primers bound to the SARS-CoV-2 S gene (Supplementary Fig. 1). Reads were aligned to a custom set of reference sequences (see Methods) using kallisto, and sample indices were corrected to a barcode whitelist. Finally, the counts of genes per sample were collated to make a sample by gene matrix (see Methods) and this was used to determine, for each sample, whether it contained viral RNA.

**Figure 2:**
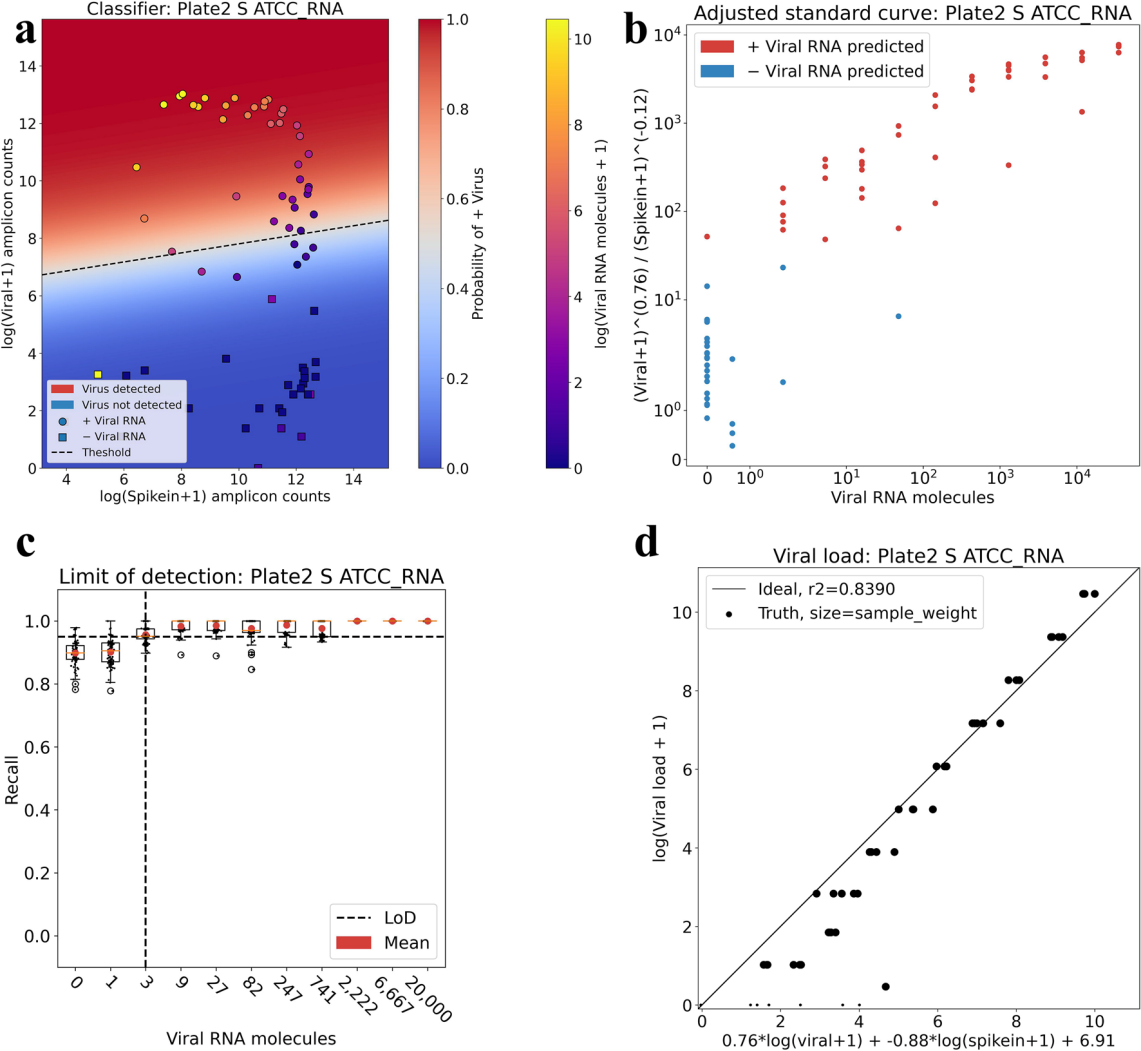
Sample classification, viral load prediction and limit of detection. (**a**) Positive and negative samples from the Plate 2 S ATCC RNA experiment can be effectively separated using logistic regression. Points correspond to samples and are colored by the known amount of viral RNA per sample. The probability of each sample having a non-zero amount of viral RNA is given by the logistic function and is painted as orthogonal to the logistic regression boundary. The shape of the point indicates whether the sample was predicted to be positive for viral RNA (circle) or negative (square). (**b**) The standard curve measuring spike-in and virus versus the known amount of viral RNA per sample with optimal exponential coefficients determined by logistic regression; samples are colored by their predicted classification. (**c**) The limit of detection as estimated from 99 rounds of split/test and logistic regression to classify samples with a non-zero amount of viral RNA. The limit of detection is defined as the number of RNA molecules for which the recall is greater than 19/20 (= 0.95) (**d**) The viral load per sample can be predicted with a weighted linear regression using the log counts from each gene. Each point is a sample, with perfect predictions lying on the diagonal line. The size of the points represents their weight, with points weighted so that each titer is represented with equal weight. The code to reproduce each figure is here: https://github.com/pachterlab/BLCSBGLKP_2020/blob/master/notebooks/diagnostic.ipynb (**a**) and (**b**), https://github.com/pachterlab/BLCSBGLKP_2020/blob/master/notebooks/lod_fda.ipynb (**c**), https://github.com/pachterlab/BLCSBGLKP_2020/blob/master/notebooks/viral_load.ipynb (**d**).

Figure 2a shows the predicted classification results for the Plate 2 S ATCC RNA experiment obtained by training a logistic regression classifier on half of the data and testing on the remaining half. The classifier learns coefficients for each covariate that optimally (by the logistic model) classify positive versus negative samples (see Methods). Crucially, the model provides a probability for each classification. Furthermore, the weights estimated in the logistic regression allow for an intuitive visualization of standard curves where virus and spike-in, suitably normalized according to regression coefficients, are measured relative to one another (Fig. 2b). This enables the assessment of the quality of a diagnostic assay in the context of classification via a standard curve.

The FDA recommends that developers of diagnostic tests assess their method using a dilution series of three replicates per concentration with inactivated virus on actual patient specimens, and then confirm the final concentration with 20 replicates^16^. Based on this guidance, the FDA defines the limit of detection (LoD) as the lowest concentration at which 19/20 replicates are positive. Therefore, to assess the LoD from a standard curve, we performed 99 replicates of the training–testing and identified the titer at which the mean recall was equal to or above 0.95 (= 19/20). The results (Fig. 2c), can be automatically generated for any HMSA for which a standard curve has been generated. Moreover, by spiking in preset amounts of virus to make a standard curve alongside a group of samples being tested, our workflow makes possible dynamic calibration of the decision boundary for groups of samples being tested together. Finally, to test the ability of kallisto|bustools to estimate the amount of virus present, we fit a linear model to the virus counts and spike-in counts. We found a strong correlation between kallisto|bustools estimates and actual viral titer (Fig. 2d). Estimation of viral load in the course of testing could help in determining time since infection^17^.

**Figure 3:**
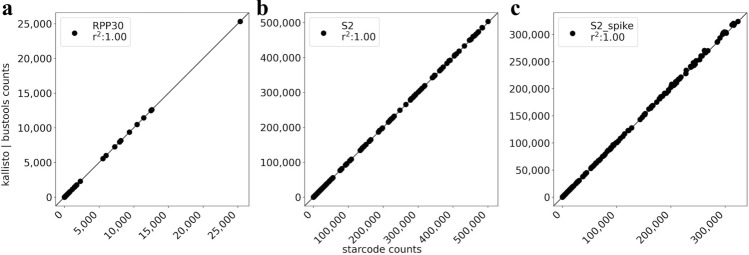
Orthogonal validation by read clustering. Scatter plots between the kallisto|bustools and the starcode workflow show near identical results on the genes targeted by the SwabSeq protocol: (**a**) RPP30, (**b**) S, and (**c**) S spike-in. Each point is a sample and the Pearson correlation is determined for the counts for a gene for all samples between kallisto|bustools and starcode. The code to reproduce this figure is here: https://github.com/pachterlab/BLCSBGLKP_2020/blob/master/notebooks/kb_v_starcode.ipynb.

To validate our results, we compared our approach to a complementary method which performed gene identification and sample index error correction using different algorithms. This alternative approach reverses the order of error correction of sample indices and assignment of biological reads to genes. First, sample indices are identified and corrected using the Illumina utility bcl2fastq. Next, reads are clustered and the number of reads in each cluster are counted using starcode^18^. The bcl2fastq + starcode approach identifies slightly fewer aligned reads but otherwise produces results that are near identical to the kallisto|bustools results (Fig. 3). However, in addition to mapping more reads, the kallisto|bustools workflow is faster and requires less memory.

Most importantly, unlike bcl2fastq, the kallisto bustools workflow is readily accessible, open source, portable, and reliable. Obtaining a copy of bcl2fastq requires a user to create an account at Illumina.com and agree to a restrictive license agreement (https://github.com/pachterlab/bcl2fastq/blob/master/bcl2fastq2-v2-20%20EULA%20(27%20Aug%202017).pdf). In addition, the behavior of kallisto bustools is transparent and well-documented, whereas the behavior of bcl2fastq is not. By generating BCL files from a set of FASTQ records and running the BCL files through bcl2fastq (code), we found that when the sample sheet used for demultiplexing has two index barcodes that are Hamming distance n apart, bcl2fastq exists with an error when the Hamming distance used for error correction is less than *n/2* + *1*. Moreover, the default Hamming distance correction for bcl2fastq 2.20 and bcl2fastq 1.8.4 differs, with 1 mismatch for the former and 0 for the latter. This issue alone can result in unwanted and unexpected behavior when using bcl2fastq.

Finally, kallisto and bustools can be easily adapted to different barcoding assays. To illustrate the point, we extended kallisto to process three different diagnostic HMSAs: covE-seq, LAMP-seq, and TRB-seq data. To validate LAMP-seq and TRB-seq, we created two synthetic sets of sequencing reads which mimic the read structure of each assay. Starting with the count matrix from the SwabSeq assay and a set of 1,000 LAMP-seq sample indices^3^ we generated 12,062,027 single-end reads consisting of a gene target and a sample index (see Methods). Similarly, we used a set of 19,200 TRB-seq sample indices^4^ to generate paired-end reads, one for the sample index and one for the target gene. In both cases, bases in each read were randomly changed to another base with a probability of 0.005 to simulate Illumina sequencing errors. We processed these reads with kallisto|bustools and obtained near identical results to those from the SwabSeq assay, thereby confirming the accuracy of the workflow for these assays (Supplementary Figs. 2 and 3). We also extended the kallisto|bustools workflow to process 2,437,573 reads produced with the covE-seq S5 protocol. The processing time was 8.17 s as compared to 20–22 h with the covE-seq mBrave and BOLD cloud platforms^14^.

## Discussion

We have demonstrated a fast, accurate, and statistically rigorous approach to process highly multiplexed sequencing assays, and have validated an intuitive and interpretable method for obtaining diagnostic results from the data. Our workflow is easily extendable to assays which target different genes of interest and assays that have a different sample index structure. In addition, our approach is extendable to assays that incorporate unique molecular identifiers (UMIs) and that target regions of genetic variation, both of which are promising future directions for HMSAs.

The SwabSeq data shows the high data quality of HMSAs and that accurate testing is technically feasible. The primary remaining challenge to widespread usage is, therefore, the organization of sample collection and associated logistics. While our work does not address the challenges of high-throughput sample collection, curation and handling of samples upstream of sequencing, our software does solve several post-sequencing logistics challenges. The low memory footprint of kallisto|bustools, specifically the requirement of less than 4 Gb of RAM (Supplementary Fig. 7) enables essentially cost-free computing in the cloud^19^. Furthermore, the speed of the workflow allows for the processing of thousands of samples within minutes (Supplementary Fig. 7), which reduces overall testing time. Moreover, the bustools software can automatically identify sample indices without the need for a pre-configured Sample Sheet, thus facilitating quality control throughout the analysis. Additionally, we made the entire workflow easily usable in the cloud via Google Colaboratory which can be used to run the workflow for free via a browser window. This should facilitate collaborative optimization of analysis workflows, rapid deployment, and will simplify analysis logistics for large-scale testing.

Finally, our software is reliable, portable, and well-documented. We can therefore be confident that, in many years from now, our preprocessing workflow will not require any overhaul or refactoring for use in future HMSAs. While we focused on SARS-CoV-2 testing in this manuscript, the methods we have developed are general and we expect that they should be applicable to future multiplexed diagnostic testing methods based on high-throughput sequencing.

## Methods

### SwabSeq

The kallisto|bustools workflow was used to process a SwabSeq experiment with two 384-well plates. The wells included each of two different SARS-CoV-2 genes, N and S, a varying amount of titered RNA from three different sources (Twist, ATCC RNA, and ATCC viral), a human gene control (RPP30), and two different lysates (HEK293, NP). The wells moreover contained barcoded primers unique to each well, synthetic RNA spike-in controls that contained the same priming regions as the target RNA from SARS-CoV-2, primers for the target SARS-CoV-2 RNA, and a one-step RT-PCR mix. Next, RT-PCR was performed on all of the wells. The wells were then pooled and sequenced on an Illumina Nextseq.

### FASTQ files

Raw BCL files were converted into FASTQ files for the kallisto|bustools workflow using ‘bcl2fastq –create-fastq-for-index-reads’ with read 1 corresponding to the Illumina i5 index, read 2 corresponding to the biological read, and index 1 corresponding to the Illumina i7 index.

### Alignment index

The genes targeted by SwabSeq were a 108 bp sequence of the SARS-CoV-2 S gene, a 6 bp modification of the SARS-CoV-2 S gene (spike-in), a 72 bp sequence of the SARS-CoV-2 N gene, a 6 bp modification of the SARS-CoV-2 N gene (spike-in), and a 65 bp sequence of the RPP30 gene (a housekeeping gene assumed to be present in all patient samples at a uniform abundance). The spike-in sequence differs from the original gene at the first 6 bp. For each spike-in/viral gene, a 10 bp window around the unique stretch of the sequence was retained and the rest of the sequence removed since any 11-mer that maps outside of the unique region could have originated from either the target gene or the spike-in. A FASTA file of all Hamming one distance variants of these target genes was made and indexed with ‘kallisto index -k 11’ with a *k*-mer length of 11.

### Read alignment and BUS file processing

A BUS file is a columnar binary file where each row is a quadruplet of a sample index, UMI, set, and count that facilitates sample index error correction and amplicon quantification. Reads from the FASTQ files generated for the kallisto|bustools workflow were pseudoaligned using ‘kallisto bus -x SwabSeq’ to generate a BUS file where all records contain the same UMI.

The BUS file was sorted with bustools sort which in addition to sorting the file lexicographically, counts and collapses the BUS records that are the same. Each half of the sample index in the BUS file was then corrected separately to a whitelist using ‘bustools correct -w whitelist.txt –split’. Correcting each half of the sample index independently is unique to SwabSeq which has a whitelist for the i5 and i7 primers, the combination of which makes the sample index. Each half of the sample index was corrected to at most Hamming distance one. The BUS file was sorted once more using ‘bustools sort’ to count and collapse any additional BUS records that are the same.

The BUS file was processed with ‘bustools count—cm’ to count the number of reads per sample that map uniquely to a specific target gene. This procedure yielded a sample by gene matrix with the number of reads. For the SwabSeq assay, this matrix consisted of 768 samples by 5 genes.

### Sample classification with logistic regression

Each sample was classified as + virus if it contained a non-zero amount of viral RNA. For each experiment, we split the data into two, half for training and half for testing, and used the log of the viral, spike-in, and RPP30 counts plus one as input. We learned the weights of a multivariate logistic regression model on the training data and used those weights to predict, for each sample, the probability that it contained virus. The logistic model used was

$$ \hat{p} = \frac{{e^{{w_{0} + w_{1} V + w_{2} K + w_{3} H}} }}{{1 + e^{{w_{0} + w_{1} V + w_{2} K + w_{3} H}} }}, $$

where *w*_*i*_ = weights, *V* = log_e_(1 + virus counts), *K* = log_e_(1 + spike-in counts) and *H* = log_e_(1 + RPP30 counts).

### Viral load prediction with weighted linear regression

We performed a weighted linear regression on the viral and spike-in counts where the samples with known zero RNA titer were weighted by one over the number of unique titers. This was done to equalize the effect of each titer in the regression. For each experiment, we split the data into two halves: a training set and a testing set. The optimal coefficients for the linear model were learned from the training data, and the viral load was then predicted using those weights for the testing data. We performed this procedure on the log_e_(1 + counts). Given the weights *w*_*i*_, the known viral load *y*_*i*_, and the log of the counts for each training sample *X*_*ij*_ plus one, the weighted linear regression model identified the vector of parameters *β* minimizing

$$ \arg \min_{\beta } \sum\limits_{i = 1}^{m} {w_{i} \left| {y_{i} - \sum\limits_{j = 1}^{n} {X_{ij} \beta_{j} } } \right|^{2} .} $$

### Limit of detection

We iteratively removed the samples corresponding to increasing amounts of RNA titer, starting with the lowest titer, and performed 99 rounds split/test and logistic regression on the remaining samples. Each run reported the recall rate, i.e. the number of true positives divided by the sum of the number of true positives and false negatives. We defined the limit of detection (LoD) as the lowest RNA titer such that the mean of the recall for that titer is greater than or equal to 19/20 (0.95).

### Validation with a complementary bcl2fastq and starcode workflow

Demultiplexed FASTQ files for the orthogonal validation were generated using the Illumina Sample Sheet and ‘bcl2fastq –no-lane-splitting –sample-sheet SampleSheet.csv’. The default with bcl2fastq is error correction of 1 mismatch, this step serves to error-correct each index separately. The resultant demultiplexed FASTQ files corresponding to each sample were clustered using ‘starcode -d2 -t1 –sphere’. Sequence centroids were assigned to genes by exact matching, and the count for each gene was given by the number of reads up to Levenshtein distance 2 away from the centroid. A sample by gene matrix was then constructed from the counts.

### Generating of reads for testing LAMP-seq and TRB-seq

Reads were generated using the sample by genes matrix from the Plate 1 HEK293 lysate N gene Twist RNA SwabSeq experiment. This matrix consisted of 96 samples and 3 targeted genes. We obtained a list of 1000 sample indices from LAMP-Seq and 19,200 sample indices from TRB-Seq as well as their associated primer sets, N1, N2, and RPP30 for TRB-seq and B_B3 for LAMP-Seq. We generated a number of reads equal to the number of amplicon counts for each sample index and target gene pair, 12,062,027 reads in total. In addition, bases in each read were randomly mutated to another base with a probability of 0.005 to simulate Illumina sequencing errors. The read structure for TRB-seq consists of paired-end reads with read 1 corresponding to a 15 bp sample index and a 22 bp constant region, and read 2 corresponding to the target gene. The read structure for LAMP-seq consists of single-end reads where the first 20 bp correspond to the targeted gene, the next 22 bp correspond to the first forward inner primer, the subsequent 10 bp correspond to the sample index, and the last 19 bp correspond to the second forward inner primer.

### LAMP-seq and TRB-seq

Reads from each assay were processed with ‘kallisto bus -x LAMPSeq’ and ‘kallisto bus -x TRBSeq’ to generate a BUS file. The BUS file was sorted with ‘bustools sort’, the sample indices were corrected to Hamming distance 1 with ‘bustools correct’ and the BUS file was sorted once more to sum duplicate records. The BUS file was then processed with ‘bustools count—cm’ to generate the technology comparisons.

### covE-seq

We downloaded data in the form of FASTQ reads for the S5 protocol^14^, and processed the reads with ‘kallisto bus -x covEseq’. The processing time for all 2,437,573 reads was determined with the time command line utility.

### bcl2fastq

Using Illumina documentation^21^ we developed a simple tool, bcltools which is available at https://github.com/pachterlab/bcltools, to generate BCL files from a set of FASTQ files. We created three FASTQ files each containing three reads. The index FASTQs contain 8 bp sequences and the biological read contains a 26 bp sequence. The I1 FASTQ sequences are all the same and the I2 FASTQ sequences differ. In the I2 FASTQ read 1 is 3 Hamming distances away from read 2 and 1 Hamming distance away from read 3. Read 2 is 2 Hamming distances away from read 3. After generating a set of BCL files, bcl2fastq 2.20 was run both with a Sample Sheet and without a Sample Sheet. When run without the Sample Sheet, we obtained the same read sequences as those that were put in. When run with the Sample Sheet, we observed that the reads were assigned to the samples in the Sample Sheet by correcting the index sequences within 1 Hamming distance.

### Data, protocol, and software availability

All the data, code and methods used to generate the results in this manuscript are open source freely available. The code to reproduce every figure and analysis for this manuscript is located here: https://github.com/pachterlab/BLCSBGLKP_2020. Each notebook can be run directly on Google Colab by pressing “Open in Colab” → “Runtime” → “Run all”. The links to all FASTQ files can be found in Supplementary Table 1.

The SwabSeq protocol is described at https://www.notion.so/Octant-SwabSeq-Testing-9eb80e793d7e46348038aa80a5a901fd.

Software programs used are listed in Supplementary Table 2.

## Supplementary information

Supplementary Information 1.

Supplementary Information 2.
