# Supplementary material for: Reliable and accurate diagnostics from highly multiplexed sequencing assays
Source: Sci Rep. 2020 Dec 10;10:21759. doi: 10.1038/s41598-020-78942-7 (PMC7730459; doi:10.1038/s41598-020-78942-7)
Supplement: Supplementary file 1 — Supplementary Information 1. [file 41598_2020_78942_MOESM1_ESM.docx]

**Supplementary Material**

Reliable and accurate diagnostics from highly multiplexed sequencing assays

A. Sina Booeshaghi^1^, Nathan B. Lubock^2^, Aaron R. Cooper^2^, Scott W. Simpkins^2^, Joshua S. Bloom^2,3^, Jase Gehring^4^, Laura Luebbert^5^, Sri Kosuri^2^, & Lior Pachter^5,6,*^

1. Department of Mechanical Engineering, California Institute of Technology, Pasadena, CA

2. Octant Inc., Emeryville, CA

3. Department of Human Genetics, University of California, Los Angeles, Los Angeles, United States

4. Department of Genome Sciences, University of Washington, Seattle, WA

5. Division of Biology and Biological Engineering, California Institute of Technology, Pasadena, CA

6. Department of Computing and Mathematical Sciences, California Institute of Technology, Pasadena, CA

*Address correspondence to lpachter@caltech.edu


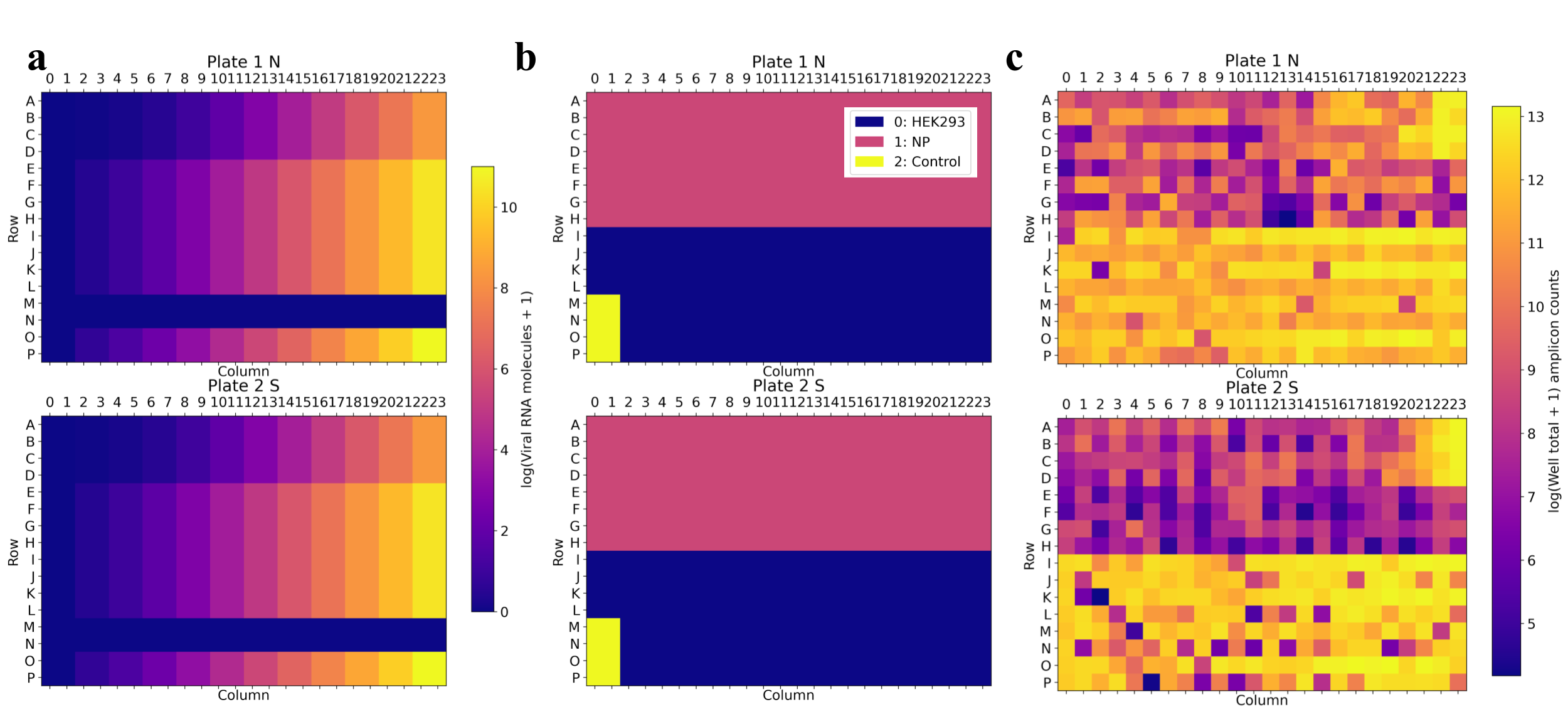


**Supplementary Figure 1: SwabSeq experimental design.** The SwabSeq experiment consisted of two 384-well plates, each with a titration series of viral RNA from two companies, Twist and ATCC, for a total of 768 uniquely barcoded samples (a). HEK293 lysate, nasopharyngeal (NP) lysate, and controls were included in all the wells of each plate (b). The first plate was used to test primers bound to the SARS-CoV-2 N gene and the second plate was used to test primers bound to the SARS-CoV-2 S gene. Samples were barcoded using the i5 and i7 Illumina indices, the former indexing the well and the latter the plate. Total amplicon counts per well were greater for HEK293 than for NP lysate (c). The code to reproduce this figure is here: [code](https://github.com/pachterlab/BLCSBGLKP_2020/blob/master/notebooks/swabseq_experimental_design.ipynb).


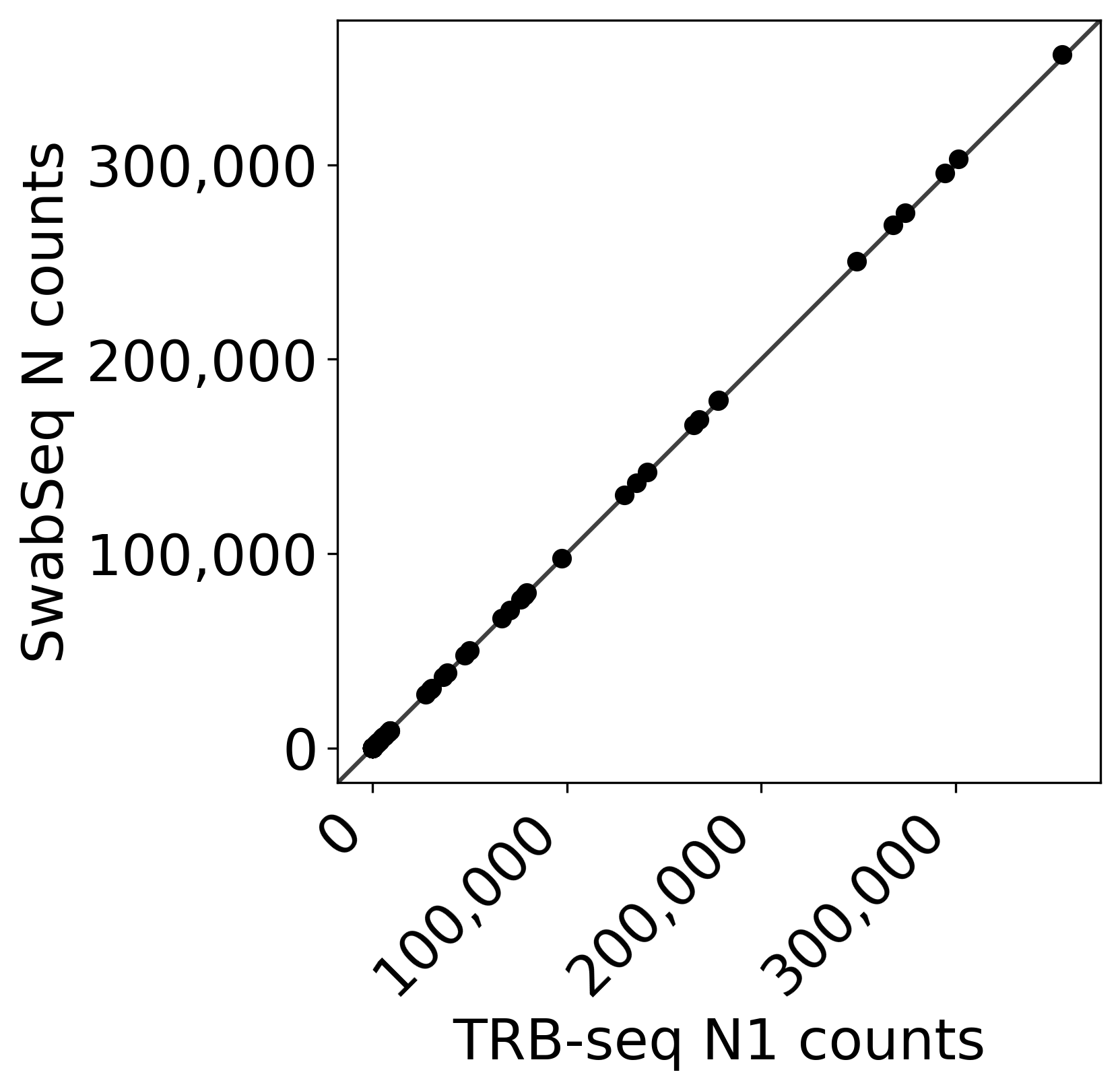


**Supplementary Figure 2:** Validation of the TRB-seq workflow with the SwabSeq workflow where each point is a sample and the counts for each sample corresponding to the N gene for SwabSeq and the N1 gene for TRB-seq are plotted. The code to reproduce this figure is here: [code](https://github.com/pachterlab/BLCSBGLKP_2020/blob/master/notebooks/trbseq.ipynb).


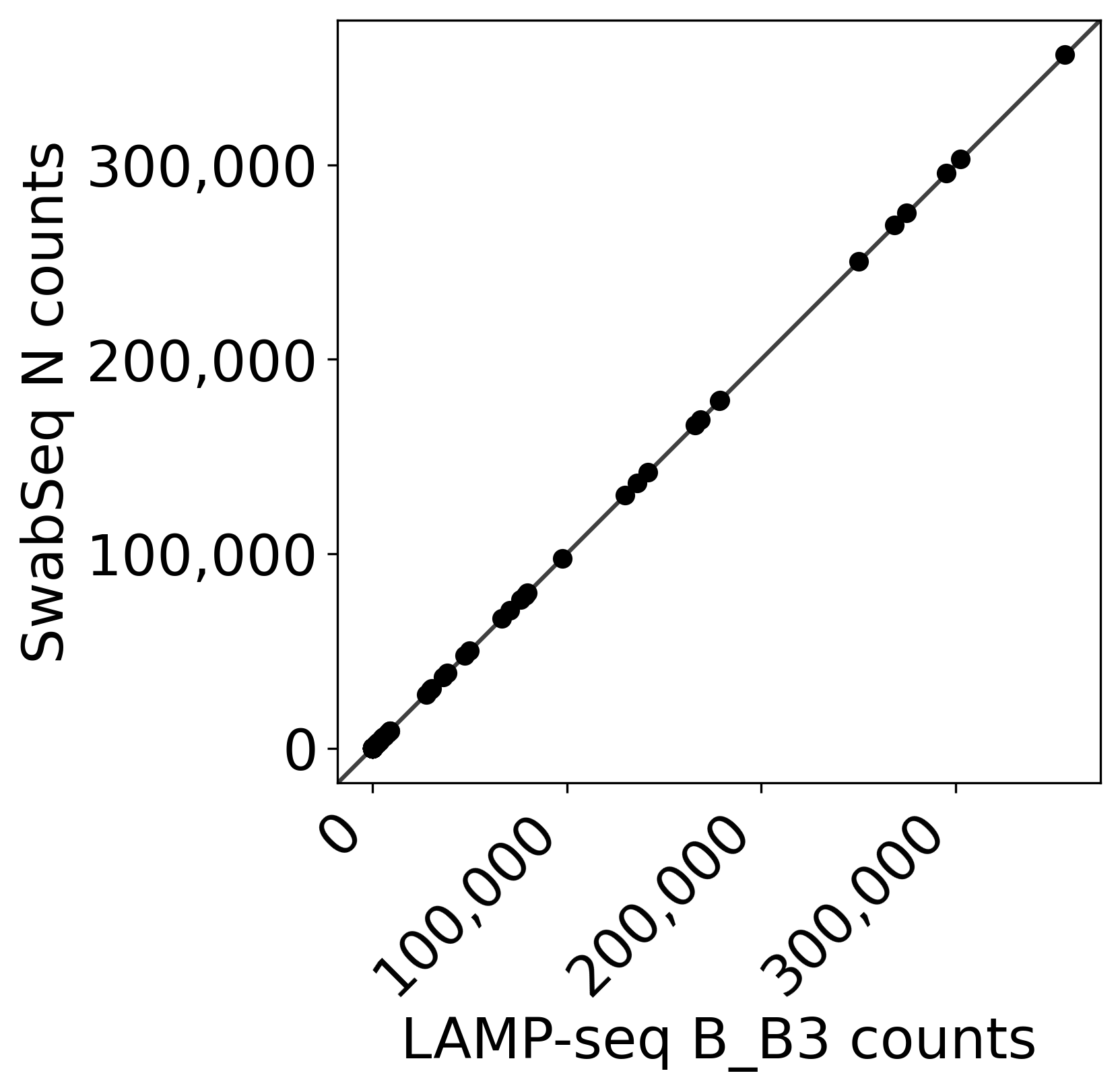


**Supplementary Figure 3:** Validation of the LAMP-seq workflow with the SwabSeq workflow, where each point is a sample. The counts for each sample corresponding to the N gene for SwabSeq and the B_B3 gene target for LAMP-seq are plotted. The code to reproduce this figure is here: [code](https://github.com/pachterlab/BLCSBGLKP_2020/blob/master/notebooks/lampseq.ipynb).

**
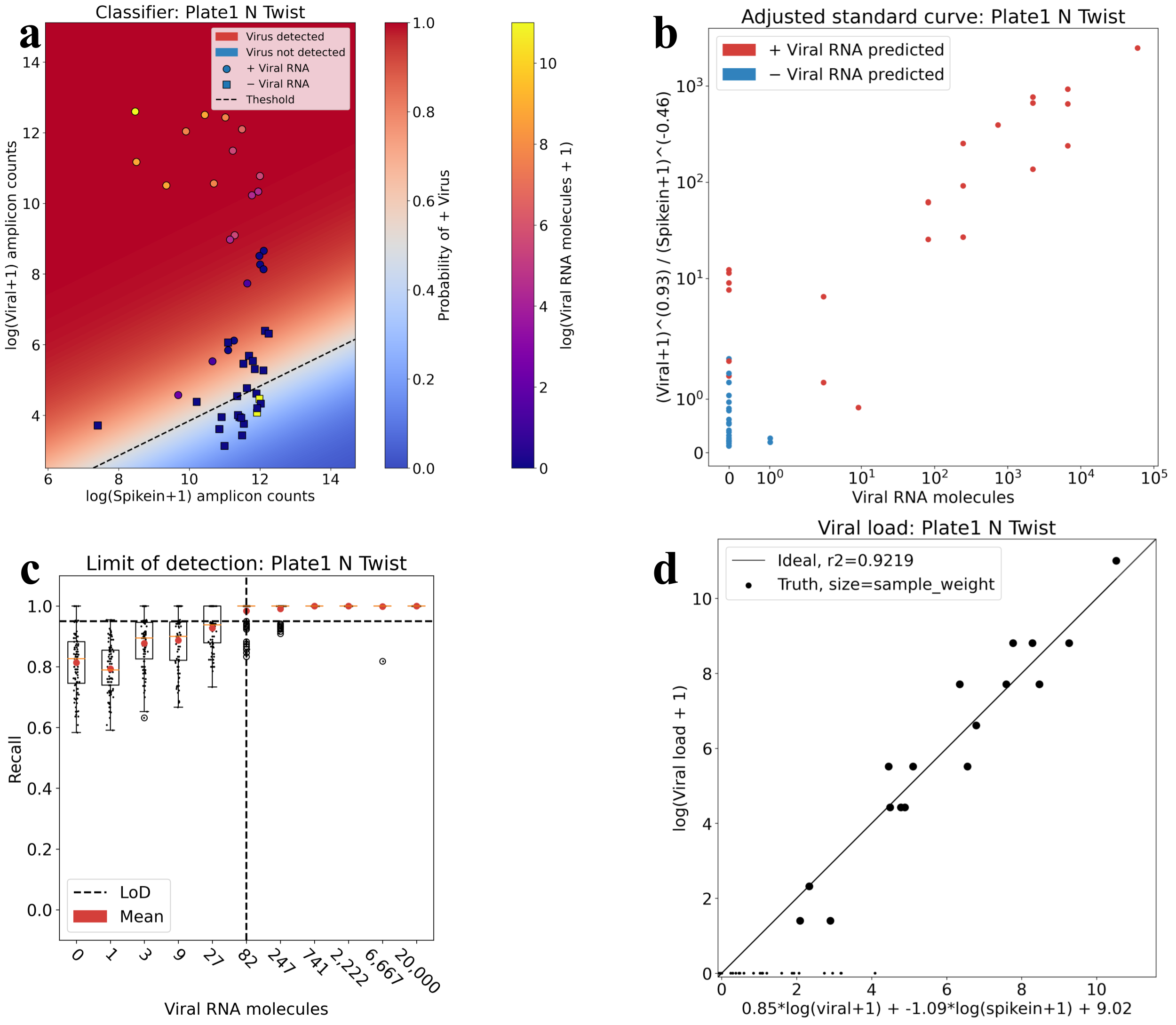
Supplementary Figure 4: Sample classification, viral load prediction and limit of detection. a)** Positive and negative samples from the Plate 1 N Twist experiment can be effectively separated using logistic regression. Points correspond to samples and are colored by the known amount of viral RNA per sample. The probability of each sample of having a non-zero amount of viral RNA is given by the logistic function and is painted as orthogonal to the logistic regression boundary. The shape of the point indicates whether it was predicted to be positive for viral RNA (circle) or negative (square). **b)** The standard curve measuring spike-in and virus vs the known amount of viral RNA per sample with optimal exponential coefficients determined by logistic regression; samples are colored by their predicted classification. **c)** The limit of detection as estimated from 99 rounds of split/test and logistic regression to classify samples with non-zero amount of viral RNA. The limit of detection is defined as the number of RNA molecules for which the recall is greater than 19/20 (=0.95) **d)** The viral load per sample can be predicted with (a weighted) linear regression using the log counts from each gene. Each point is a sample, with perfect predictions lying on the diagonal line. Size of points represents their weight, with points weighted so that each titer is represented with equal weight. The code to reproduce each figure is here: [code (a) and code (b)](https://github.com/pachterlab/BLCSBGLKP_2020/blob/master/notebooks/diagnostic.ipynb), [code (c)](https://github.com/pachterlab/BLCSBGLKP_2020/blob/master/notebooks/lod_fda.ipynb), [code (d)](https://github.com/pachterlab/BLCSBGLKP_2020/blob/master/notebooks/viral_load.ipynb).

**
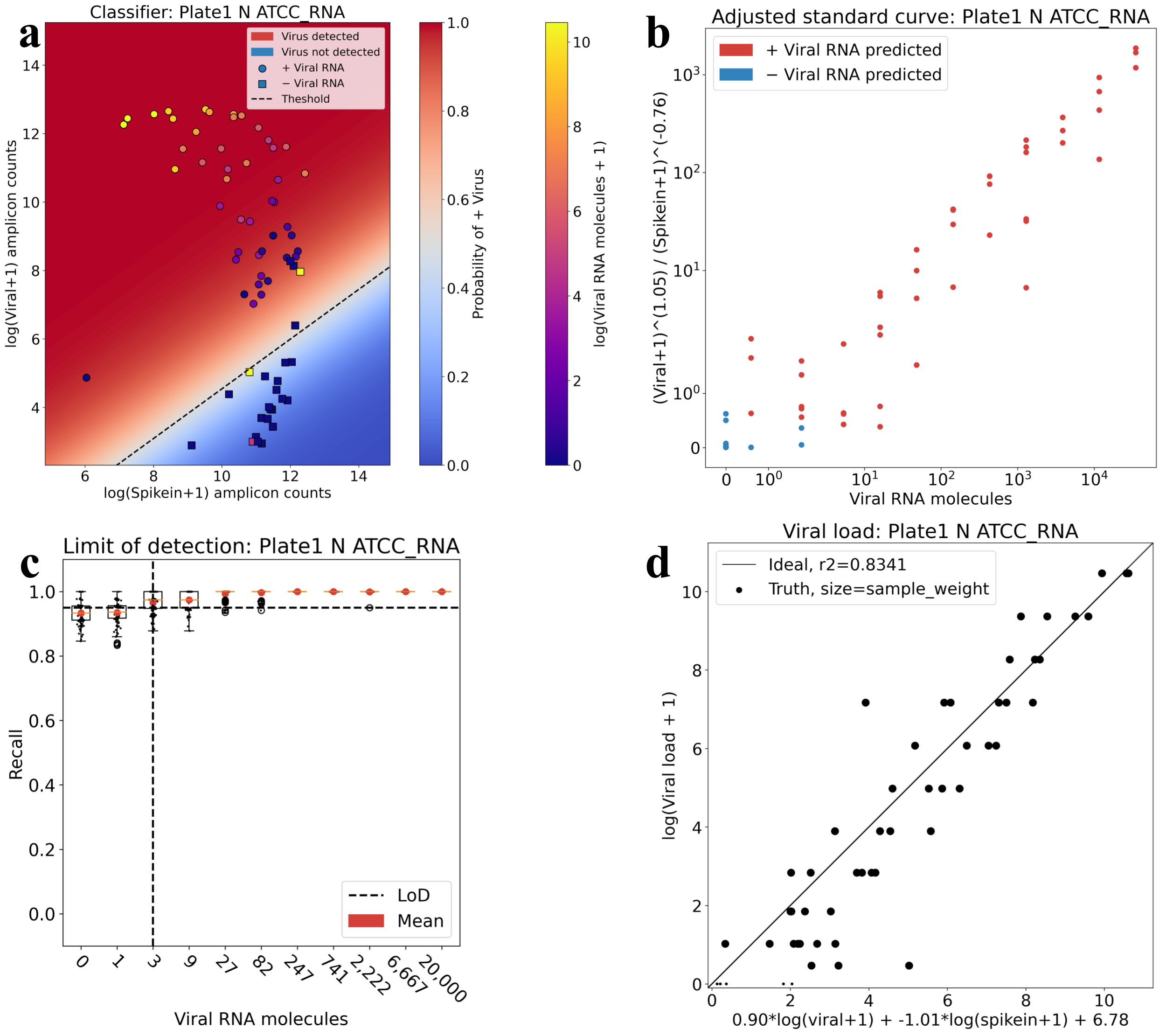
**

**Supplementary Figure 5: Sample classification, viral load prediction and limit of detection. a)** Positive and negative samples from the Plate 1 N ATCC RNA experiment can be effectively separated using logistic regression. Points correspond to samples and are colored by the known amount of viral RNA per sample. The probability of each sample of having a non-zero amount of viral RNA is given by the logistic function and is painted as orthogonal to the logistic regression boundary. The shape of the point indicates whether it was predicted to be positive for viral RNA (circle) or negative (square). **b)** The standard curve measuring spike-in and virus vs the known amount of viral RNA per sample with optimal exponential coefficients determined by logistic regression; samples are colored by their predicted classification. **c)** The limit of detection as estimated from 99 rounds of split/test and logistic regression to classify samples with non-zero amount of viral RNA. The limit of detection is defined as the number of RNA molecules for which the recall is greater than 19/20 (=0.95) **d)** The viral load per sample can be predicted with (a weighted) linear regression using the log counts from each gene. Each point is a sample, with perfect predictions lying on the diagonal line. Size of points represents their weight, with points weighted so that each titer is represented with equal weight. The code to reproduce each figure is here: [code (a) and code (b)](https://github.com/pachterlab/BLCSBGLKP_2020/blob/master/notebooks/diagnostic.ipynb), [code (c)](https://github.com/pachterlab/BLCSBGLKP_2020/blob/master/notebooks/lod_fda.ipynb), [code (d)](https://github.com/pachterlab/BLCSBGLKP_2020/blob/master/notebooks/viral_load.ipynb).

**
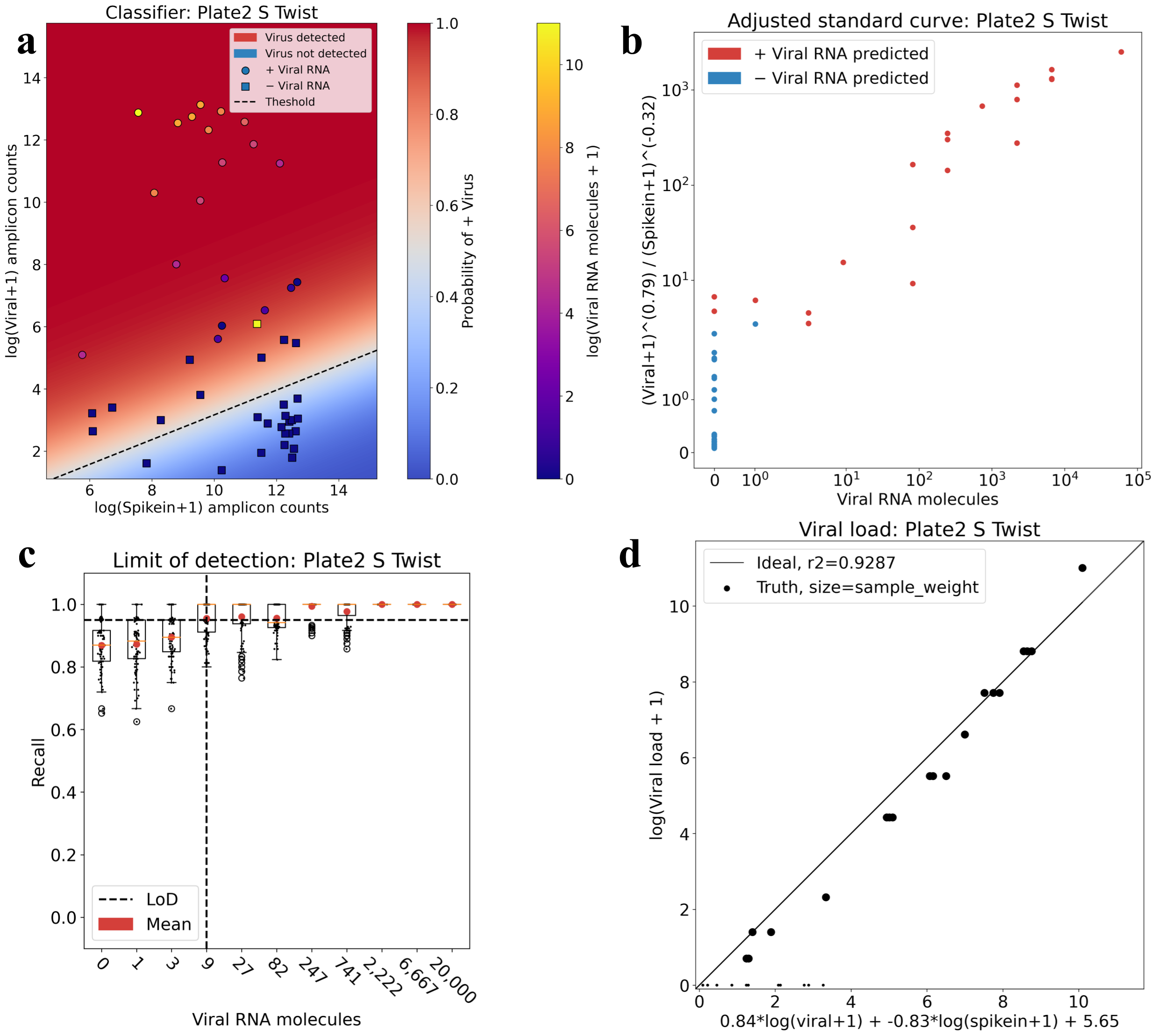
**

**Supplementary Figure 6: Sample classification, viral load prediction and limit of detection. a)** Positive and negative samples from the Plate 2 S Twist experiment can be effectively separated using logistic regression. Points correspond to samples and are colored by the known amount of viral RNA per sample. The probability of each sample of having a non-zero amount of viral RNA is given by the logistic function and is painted as orthogonal to the logistic regression boundary. The shape of the point indicates whether it was predicted to be positive for viral RNA (circle) or negative (square). **b)** The standard curve measuring spike-in and virus vs the known amount of viral RNA per sample with optimal exponential coefficients determined by logistic regression; samples are colored by their predicted classification. **c)** The limit of detection as estimated from 99 rounds of split/test and logistic regression to classify samples with non-zero amount of viral RNA. The limit of detection is defined as the number of RNA molecules for which the recall is greater than 19/20 (=0.95) **d)** The viral load per sample can be predicted with (a weighted) linear regression using the log counts from each gene. Each point is a sample, with perfect predictions lying on the diagonal line. Size of points represents their weight, with points weighted so that each titer is represented with equal weight. The code to reproduce each figure is here: [code (a) and code (b)](https://github.com/pachterlab/BLCSBGLKP_2020/blob/master/notebooks/diagnostic.ipynb), [code (c)](https://github.com/pachterlab/BLCSBGLKP_2020/blob/master/notebooks/lod_fda.ipynb), [code (d)](https://github.com/pachterlab/BLCSBGLKP_2020/blob/master/notebooks/viral_load.ipynb).

**
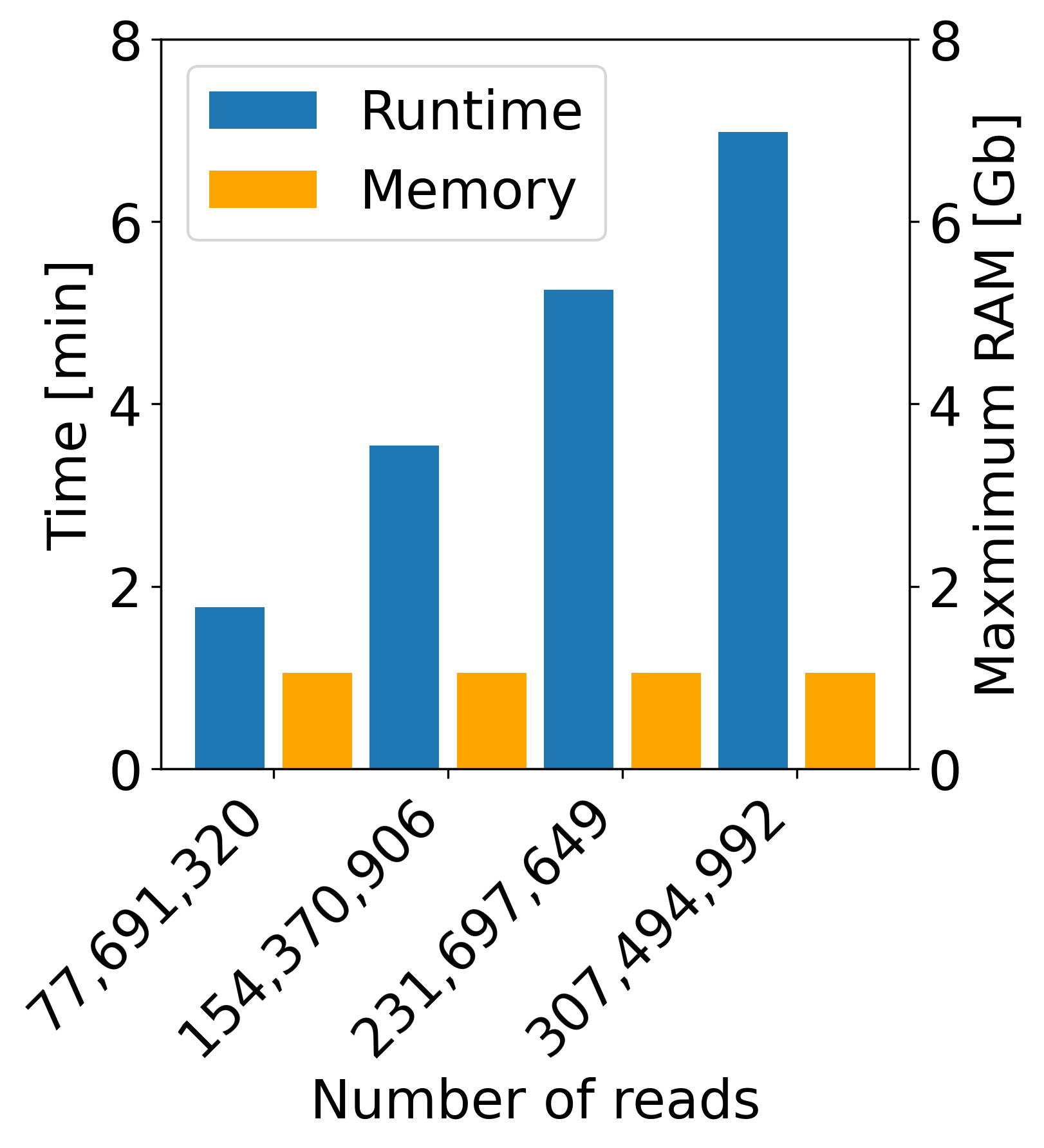
**

**Supplementary Figure 7:** Runtime and memory footprint of kallisto | bustools shown for a total of 1 lane (77,691,320 reads), 2 lanes (154,370,906 reads), 3 lanes (231,697,649 reads), and 4 lanes (307,494,992 reads) of Illumina NextSeq 550 data. The code to reproduce this figure is here: [code](https://github.com/pachterlab/BLCSBGLKP_2020/blob/master/notebooks/memtime.ipynb).

| **Lane** | **Index 1** | **Read 1** | **Read 2** |
| --- | --- | --- | --- |
| 1 | [Lane 1 Index 1](https://caltech.box.com/shared/static/3i46orxgtwlaho7f9z255hplg6tvfs6h.gz) | [Lane 1 Read 1](https://caltech.box.com/shared/static/0f3h3837xvo2dcqkax67njops5s4zxz0.gz) | [Lane 1 Read 2](https://caltech.box.com/shared/static/lh0nyo1v95k1s7nvw4zj84yl6jwx3hpg.gz) |
| 2 | [Lane 2 Index 1](https://caltech.box.com/shared/static/rxb4h3owka0x2deh0royge4w55u0bub5.gz) | [Lane 2 Read 1](https://caltech.box.com/shared/static/orqpywdlryss9df49tha4i8yywlswrtj.gz) | [Lane 2 Read 2](https://caltech.box.com/shared/static/2eyqb989cohgv4h00mtjj3lrn3tpgi41.gz) |
| 3 | [Lane 3 Index 1](https://caltech.box.com/shared/static/0r5ezocuh9mzxxj6nsf1fgfl38fdbfye.gz) | [Lane 3 Read 1](https://caltech.box.com/shared/static/7q3xgu2lp2t46638c1rg569duz5kdw9a.gz) | [Lane 3 Read 2](https://caltech.box.com/shared/static/d48e56j9qqxo4sveqiwa3lq9bwzxua4f.gz) |
| 4 | [Lane 4 Index 1](https://caltech.box.com/shared/static/pkgyve9ft7u09du66a0e3r4a3ae4mmhc.gz) | [Lane 4 Read 1](https://caltech.box.com/shared/static/krcntl56mgt91ca08qvljfhohh9g197m.gz) | [Lane 4 Read 2](https://caltech.box.com/shared/static/nvfmriwe1891lfqrvedmoko6i5sd0mm6.gz) |

**Supplementary Table 1:** Links to the SwabSeq FASTQ files analyzed.

| **Software** | **Version** |
| --- | --- |
| [Anndata](https://github.com/theislab/anndata) | 0.7.1 |
| [bustools](https://github.com/BUStools/bustools/) | 0.40.0 (branch: covid) |
| [awk (GNU awk)](https://www.gnu.org/software/gawk/manual/gawk.html) | 4.1.4 |
| [grep (GNU grep)](https://www.gnu.org/software/grep/manual/grep.html) | 3.1 |
| [kallisto](https://github.com/pachterlab/kallisto) | 0.46.2 (branch: covid) |
| [kb_python](https://github.com/pachterlab/kb_python) | 0.24.4 (branch: count-kite) |
| [Matplotlib](https://github.com/matplotlib/matplotlib) | 3.0.3 |
| [Numpy](https://github.com/numpy/numpy) | 1.18.1 |
| [Pandas](https://github.com/pandas-dev/pandas) | 0.25.3 |
| [Scipy](https://github.com/scipy/scipy) | 1.4.1 |
| [sed (GNU sed)](https://www.gnu.org/software/sed/) | 4.4 |
| [sklearn](https://github.com/scikit-learn/scikit-learn) | 0.22.1 |
| [starcode](https://github.com/gui11aume/starcode) | v1.3 17-07-2018 |
| [tar (GNU tar)](https://www.gnu.org/software/tar/) | 1.29 |
| [time (GNU time)](http://man7.org/linux/man-pages/man1/time.1.html) | 1.7 |

**Supplementary Table 2:** Software versions used.
