# Supplementary material for: Reliable and accurate diagnostics from highly multiplexed sequencing assays
Source: Sci Rep. 2020 Dec 10;10:21759. doi: 10.1038/s41598-020-78942-7 (PMC7730459; doi:10.1038/s41598-020-78942-7)
Supplement: Supplementary file 2 — Supplementary Information 2. [file 41598_2020_78942_MOESM2_ESM.docx]

**Supplementary Note**

Reliable and accurate diagnostics from highly multiplexed sequencing assays

A. Sina Booeshaghi^1^, Nathan B. Lubock^2^, Aaron R. Cooper^2^, Scott W. Simpkins^2^, Joshua S. Bloom^2,3^, Jase Gehring^4^, Laura Luebbert^5^, Sri Kosuri^2^, & Lior Pachter^5,6,*^

1. Department of Mechanical Engineering, California Institute of Technology, Pasadena, CA

2. Octant Inc., Emeryville, CA

3. Department of Human Genetics, University of California, Los Angeles, Los Angeles, United States

4. Department of Genome Sciences, University of Washington, Seattle, WA

5. Division of Biology and Biological Engineering, California Institute of Technology, Pasadena, CA

6. Department of Computing and Mathematical Sciences, California Institute of Technology, Pasadena, CA

*Address correspondence to lpachter@caltech.edu

To analyze diagnostic testing sequence data we have adapted two programs, kallisto and bustools, to perform all the necessary processing steps: **1) Create an index, 2) align the reads with kallisto, 3) sort the BUS file with bustools sort, 4) correct the barcodes with bustools correct and a whitelist, 5) sort the BUS file again, 6) count the multiplicity for each BUS record to generate a sample by gene matrix.** The workflow is designated kallisto | bustools, where the pipe ( | ) between the tools highlights the fact that results from kallisto are passed onto bustools in the workflow:

1. **An “index”, a compressed data structure based on a de Bruijn graph**[^1,2^](https://paperpile.com/c/jYHIKg/rMeS+zkAd)**, is constructed from the gene sequences that are targeted in the diagnostic test.** These gene sequences may consist of human sequences, viral sequences, or synthetic sequences. The index is constructed with kallisto using the kallisto index option. The input consists of the gene sequences, or relevant parts of them, in FASTA format.
2. **Raw sequence data in FASTQ format is aligned and output to a BUS file using kallisto.**kallisto is called with an option specific to the diagnostic technology. For example, for SwabSeq, kallisto is called with kallisto -x SwabSeq. This specifies the location of the sample indices in the read files, along with the location of the biological sequence. It is straightforward to add new technologies (using -x) to kallisto. kallisto processes the FASTQ records one at a time. When processing a record, kallisto first aligns the biological sequence to the genes, and results of the alignment are stored in the third column of the BUS file. The alignment relies on *k-*mer matching; a biological read can be identified as originating from a gene in the reference as long as there is a unique match of a substring of length *k*. This makes the alignment robust to mismatches resulting from sequencing errors, as well as insertions and deletion. The second column of the BUS file is reserved for storing a unique molecular identifier for the record; currently it is unused for this application and is filled with a dummy “A”. If the biological sequence associated with a sample index aligns to the kallisto index, then the index is copied into the first column of the BUS file. In the case of SwabSeq, the sample index is split between reads and kallisto automatically handles this thanks to the specification with the -x SwabSeq option in kallisto. The two separate indices are merged into one barcode in the BUS file. Once kallisto has finished processing the reads, the resultant BUS file contains a table where each record corresponds to a sequence obtained from a sample, and where the record contains information about its sample index and gene (which the biological sequence corresponding to that record aligned to). This BUS file is ready to be processed with various bustools programs.
3. **bustools sort is run to sort the BUS file by barcode sequence.** This step is key to ensuring that all subsequent bustools commands run in constant memory, and processing time scales linearly with the number of BUS records. A memory limit can be set for this step; in this work we use a maximum of 1Gb RAM.
4. **bustools correct is used to correct errors in the barcodes in the BUS file.** This is done using a “whitelist” of barcodes from a sample sheet describing the diagnostic experiment. If whitelist barcodes have been designed to be at least 3 mismatches apart from each other (Hamming distance 3)[^3,4^](https://paperpile.com/c/jYHIKg/Jtuk+bAcb), then a single mismatch can be corrected. The bustools correct command will correct all barcodes within 1 mismatch (Hamming distance 1) of a whitelist barcode. Alternatively, the bustools whitelist command can be run before bustools correct to automatically identify valid barcodes (using the total counts associated with each barcode). The resultant whitelist can then be used with the bustools correct command to error correct the remaining barcodes.
5. **bustools sort is run again.** The purpose of this sort command is to collate the counts of genes associated with each sample index. These counts are recorded in a fourth column of the BUS file, indicating multiplicity of the record.
6. **bustools count is used to produce a human readable table of sample barcode sequences along with the counts of genes detected for each sample.**

## References

1. [Compeau, P. E. C., Pevzner, P. A. & Tesler, G. How to apply de Bruijn graphs to genome assembly. *Nat. Biotechnol.* **29**, 987–991 (2011).](http://paperpile.com/b/jYHIKg/rMeS)

2. [Bray, N. L., Pimentel, H., Melsted, P. & Pachter, L. Near-optimal probabilistic RNA-seq quantification. *Nat. Biotechnol.* **34**, 525–527 (2016).](http://paperpile.com/b/jYHIKg/zkAd)

3. [Ashlock, D., Houghten, S. K., Brown, J. A. & Orth, J. On the synthesis of DNA error correcting codes. *Biosystems.* **110**, 1–8 (2012).](http://paperpile.com/b/jYHIKg/Jtuk)

4. [Conway, J. & Sloane, N. Lexicographic codes: Error-correcting codes from game theory. *IEEE Trans. Inf. Theory* **32**, 337–348 (1986).](http://paperpile.com/b/jYHIKg/bAcb)
